# BMD in Transient Osteoporosis and Bone Marrow Edema Syndrome: A Scoping Review

**DOI:** 10.1155/joos/9976282

**Published:** 2026-04-02

**Authors:** Eivind Hasvik, Anne Julsrud Haugen, Lars Grøvle

**Affiliations:** ^1^ Department of Physical Medicine and Rehabilitation, Østfold Hospital Trust, Grålum, Norway, sykehuset-ostfold.no; ^2^ Department of Rheumatology, Østfold Hospital Trust, Grålum, Norway, sykehuset-ostfold.no

## Abstract

Transient osteoporosis of the hip (TOH), regional migratory osteoporosis (RMO), and bone marrow edema syndrome (BMES) are increasingly recognized as manifestations of the same underlying condition. Hallmark features include joint pain, bone marrow edema on MRI, and localized demineralization. However, densitometry data in this context are fragmented and difficult to interpret. This study screened 561 publications and identified 188 patients with quantifiable bone mineral density (BMD) data—*Z*‐, *T*‐scores (for patients under 50), or raw values—obtained during active disease and unaffected by treatment. Mixed‐effects modeling and linear regression showed significant BMD reductions in symptomatic hips: marginal mean *Z*/*T*‐scores of −2.12 (95% CI −2.4 to −1.88, *n* = 48). In pairwise analyses, symptomatic hips had lower BMD than asymptomatic hips: marginal mean Z/T‐score difference −1.2 (95% CI −1.7 to −0.8, *n* = 16) and raw score difference of −0.173 g/cm^2^ (21% lower; 95% CI −0.214 to −0.132, *n* = 19). Despite few reports of back pain, spine BMD was also reduced: mean *Z*/*T*‐score of −1.83 (95% CI −2.1 to −1.6, *n* = 65). Among perinatal women, spine BMD reached −2.2 (95% CI −2.6 to −1.8, *n* = 22). No BMD differences emerged between osteoporosis‐ and edema‐related terminology groups, supporting a shared disease mechanism.

## 1. Introduction

The terms transient osteoporosis of the hip (TOH), regional migratory osteoporosis (RMO), and bone marrow edema syndrome (BMES) have all been used to describe patients presenting with spontaneous pain in a lower limb joint accompanied by demineralization and bone marrow edema when no other underlying cause is identified.

The earliest term introduced was TOH, which referred to patients with hip pain and radiographic evidence of proximal femur demineralization [1]. Subsequently, it became evident that similar symptoms could occur in the knee, ankle, and foot—sometimes migrating between joints or recurring—leading to the term RMO [2]. Historically, these diagnoses relied on subjective assessments of bone demineralization observed on radiographs, a method now recognized as unreliable. The term BMES was introduced in the 1980s following the advent of MRI, which enabled the detection of bone marrow edema in affected joints [3, 4]. However, none of these diagnostic terms were formally defined, and a variety of other expressions—such as “recurrent migratory transient bone marrow edema” or “migrating transient osteoporosis of the hip”—also entered common usage. Through a comprehensive review of the literature, we previously identified numerous undefined and ambiguous diagnostic terms, in addition to TOH, RMO, and BMES [5]. A subsequent scoping review demonstrated that the demographic and clinical characteristics of patients described under these various labels were broadly similar, suggesting a common pathology across these terminologies [6]. The primary symptom was spontaneous pain, most often in the hip, which typically resolved within 4–6 months but sometimes recurred in the same or another lower limb joint. Signs of bone marrow edema and radiographic demineralization also tended to resolve spontaneously. The average age of onset was approximately 40 years, with men and women equally affected. Many of the women were pregnant or postpartum, with hip fractures reported in some.

Although osteoporosis has long been considered a hallmark of these entities, available data on bone mineralization are surprisingly sparse. Their relative rarity and inconsistent terminology have likely contributed to the fact that no systematic summary regarding bone mineral density (BMD) exists. Whether BMD in patients reported under osteoporosis‐related terminology, such as TOH and RMO, differs from those categorized under bone marrow edema‐related terminology, such as BMES, remains unresolved. A key challenge is the limited applicability of the standard reference method—dual‐energy x‐ray absorptiometry (DXA)—outside the lumbar spine and proximal femur. Consequently, it cannot routinely evaluate BMD in affected bones in patients with knee, ankle, or foot involvement. Such patients constitute approximately one‐third of the total population [6].

The aetiology of this intriguing condition remains unknown, and its pathophysiology is poorly characterized. Advancing knowledge of bone metabolism is critical to improving our comprehension of the disease and guiding the establishment of a unified diagnosis.

The present study is part of a larger project that aims to deepen the insight into this condition through a critical review of the existing literature. The specific objective here is to perform a scoping review to explore BMD data reported in patients designated with TOH, RMO, BMES, or similar terms.

## 2. Materials and Methods

**FIGURE 1:**
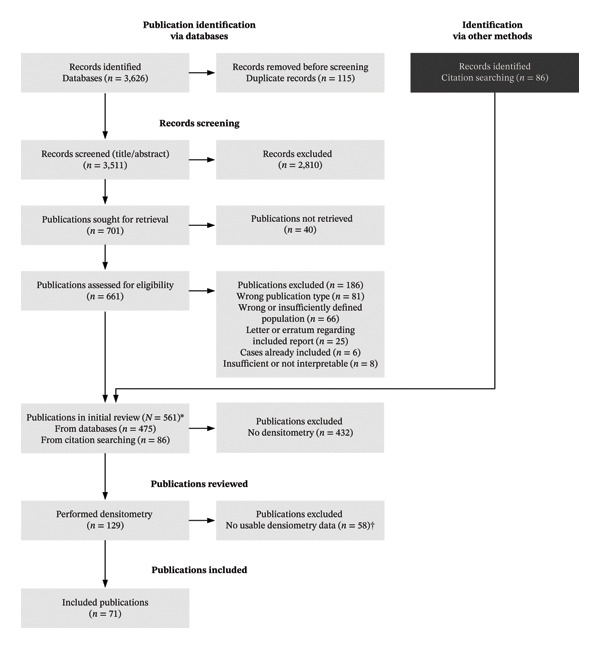
Flow diagram illustrating the systematic review process for identifying and including studies reporting bone mineral density (BMD) data. ^∗^Details of the search methodology and the data extraction process were previously described [5, 6]. ^†^See complete list of exclusion criteria in methods.

This study was designed as a scoping review, an approach suited to identifying and mapping key characteristics of a concept in areas with inconsistent terminology or limited prior synthesis [7, 8]. It draws on data from a literature search conducted as part of a larger project on TOH, RMO, BMES, and similar conditions; the search methodology and the data extraction process have been described in detail in our prior publications [5, 6]. The Embase and MEDLINE databases on the Ovid platform (Wolters Kluwer, New York City, NY) were searched from inception to 16 February 2023. This search identified 561 publications, encompassing a total of 2924 patients. Eligibility criteria were formulated using the SPIDER tool [9], and the search strategy was developed following the PRESS checklist [10]. The study protocol was preregistered on the Open Science Framework [11]. During data extraction, we recorded whether the publications mentioned the use of bone densitometry. Of the 561 publications, 432 did not report on densitometry and were excluded, leaving 129 publications containing some form of densitometry data, which were forwarded for further review (see Figure 1). The reporting of this scoping review adheres to the PRISMA‐ScR guidelines [12] (Supporting 4.0).

## 3. Data Extraction

### 3.1. BMD Variables

We extracted BMD data obtained by DXA, quantitative computed tomography (QCT), single‐photon/dual‐photon absorptiometry (SPA/DPA), and ultrasound (US). To evaluate effects on affected bone, we extracted BMD data from symptomatic locations. To evaluate effects on nonaffected bone, we extracted data from contralateral asymptomatic locations and the lumbar spine (L1–L4) when available. For the hips, we extracted data from the femoral neck and the total hip. The total hip measurement encompasses the femoral neck, trochanter, and intertrochanteric region. If the specific hip measurement site was not specified, we recorded the data as “unspecified hip”. If both hips were symptomatic, data from the hip with the lowest BMD were recorded.

To ensure comparability, we prioritized recording BMD *Z*‐scores, which represent the number of standard deviations (SDs) by which an individual’s BMD differs from the mean value expected for their age and sex [13]. Some studies reported BMD as percentages of the expected mean (PEM) for age and sex (Z‐PEMs). For patients under 50 years of age without Z‐ or Z‐PEM scores, we recorded T‐scores, which indicate the number of SDs by which BMD deviates from the mean value in young, healthy individuals [13]. Before age 50, bone density is relatively stable, and T‐scores diverge minimally from *Z*‐scores, making them a suitable proxy [14]. Alternatively, we recorded PEM for young adults (T‐PEMs). If none of these standardized measures were available, we recorded bone mineral raw scores (g/cm^2^) from symptomatic and contralateral asymptomatic locations. The purpose of recording raw scores was to analyze intraindividual BMD differences between affected and nonaffected bone.

We exclusively recorded numerical BMD scores. Semiquantitative or verbal descriptions, such as “normal” or “osteoporosis”, as well as data reported only as being above or below certain thresholds, were excluded. We also excluded measurements where the measured locations were not specified, the densitometry modality was not provided or the measurements were conducted after initiation of bone‐active drugs, except for calcium and vitamin D supplementation.

We recorded BMD measurements taken while patients had symptoms and during follow‐up. Measurements solely performed after symptom resolution and follow‐up data obtained beyond 5 years after the initial episode were excluded. Measurements in children (< 15 years) were also excluded.

Depending on the format reported in the publications, we recorded BMD data either as individual‐level data when BMD values were specified for single patients or as aggregate‐level data when only summary statistics for groups were reported.

Data were independently extracted by two reviewers. Discrepancies were resolved by discussion or decided by a third reviewer. When necessary, text was translated using Google Translate [15], and optical character recognition was performed. Data available only as figures were extracted using WebPlotDigitizer 5.0 (Automeris LLC, Pacifica, CA) [16].

### 3.2. Background Variables

We utilized previously extracted and reported data [5, 6] on age, sex, and the occurrence of symptoms in relation to the perinatal period, defined here as pregnancy and 6 months postpartum. Additionally, we used data on the location and duration of symptoms, imaging modalities employed, abnormal imaging findings, and clinical fractures. We did not classify subchondral lesions on MRI reported as fracture lines or microfractures as clinical fractures.

### 3.3. Analyses

No hypothesis was specified for this study; the analyses are descriptive only. Discrete or categorical data were summarized using counts and percentages. Numerical continuous data were summarized using mean and SD or 95% confidence interval (CI), grouped mean and SD [17], or median and interquartile range (IQR). Normality of BMD *Z*‐ and *T*‐scores was assessed through visual inspection of Q‐Q and probability density plots, as well as statistical testing.

Hip BMD marginal means, stratified by sex and side (symptomatic or asymptomatic), were estimated post hoc from regression analysis. All measurements from an individual were used. A mixed‐effects model was applied to account for the correlation between multiple hip measurement sites, with adjustment for perinatal status, side (asymptomatic or symptomatic), and whether BMD was reported using *T*‐ or *Z*‐scores. Spine BMD marginal means, stratified by sex, were estimated post hoc from linear regression analysis, adjusted for perinatal status and whether BMD was reported using *T*‐ or *Z*‐scores.

Similarly, in patients with BMD values from both asymptomatic and symptomatic hips, marginal means and the marginal mean difference were estimated post hoc from regression analysis. A mixed‐effects model regression accounted for the correlation between multiple hip measurement sites, as all measures from an individual were used. The model was adjusted for side (asymptomatic or symptomatic) and whether BMD was reported using *T*‐ or *Z*‐scores. Marginal means and the marginal mean difference in raw BMD scores were estimated from a linear regression model, adjusted for side, sex, and age. These differences were visualized using Gardner–Altman plots [18], including error density curves derived by bootstrapping with 5000 bias‐corrected and accelerated resamples to assess the variability in the estimated differences [19].

In all regression models, *Z*‐scores were preferred over *T*‐scores if available. Only *T*‐scores for patients aged < 50 years were included in the models [14]. Models were assessed for regression assumptions by generating simulated standardized residuals and testing these for uniformity and dispersion [20]. The marginal means, along with their 95% CIs, were estimated with weights based on the frequency of the stratified categorical predictor values in the observed data, which ensures that the estimates reflect the distribution of the data [21].

The analyses were performed using the R programming language v4.4.2 (2024‐10‐31) (R Foundation for Statistical Computing, Vienna, Austria) and RStudio software environment 2024.12.0 + 467 (Posit Software, PBC, Boston, MA), the R packages *DHARMa* 0.4.7 [20], *lme4* v1.1‐36 [22], *marginaleffects* v0.24.0 [21], *rsample* v1.2.1 [23], and *tidyverse* v2.0.0 [24].

## 4. Results

Among the 129 publications selected for review (see Figure 1), 114 provided individual‐level data, and 15 reported aggregate BMD data, on a total of 376 patients. From these publications, 188 patients were included in the present study, while the remaining were excluded based on preset criteria shown in Table 1. The included studies were published between 1990 and 2022 and comprised 112 patients with individual‐level data (68 publications) and 76 patients with aggregate‐level data (3 publications) (Supporting 3.1–3). Among the 188 patients, women comprised 71% of those with reported sex, and about 31% were perinatal. More patients were categorized with osteoporosis‐related terminology (*n* = 108) compared to bone marrow edema‐related terminology (*n* = 80) (Supporting 1.0).

**TABLE 1:** Number of patients who were reported to have undergone densitometry who were excluded based on preset criteria.

Exclusion criteria	Patients, *n* ^a^
Measured after symptom resolution	30
Measured after initiation of bone‐active drugs^b^	6
Measurement site not specified	3
No BMD data reported	88
No numerical BMD data reported	27
Age ≥ 50 and reported *T*‐score	25
Unknown densitometry modality	1
Child	1
Symptomatic or asymptomatic locations not specified	4
Assumed data‐error	3

^a^In some studies, the authors did not provide explicit details regarding how many patients had a BMD assessment. In these instances, all study patients were excluded.

^b^Alendronate (*n* = 2), Pamidronate (*n* = 1), Calcitonin (*n* = 1), Glucocorticoids (*n* = 1), Estradiol/medroxyprogesterone (*n* = 1).

The clinical characteristics of the patients with individual‐level data are shown in Table 2. The median (IQR) age of the sample with individual‐level data were 36 (32‐44) (*n* = 109), years and 51% (57/112) were women. Of the women with individual‐level data, 35 (61%) were perinatal. Almost all patients had pain located in the hip, *n* = 104 (93%).

**TABLE 2:** Clinical characteristics of patients with individual‐level data (*n* = 112).

Age, median (IQR)	36 (32–44) (*n* = 109) (%)
Women, *n* (%)	57 (51)
Perinatal (% of women)	61 (35/57)

Pain location, *n* (%)	
Hip	104 (93)
Knee	17 (15)
Ankle	8 (7)
Foot	6 (5)
Spine	3 (3)
Gluteal	1 (1)
Number of episodes, *n* (%)^a^	

1 episode	87 (78)
2 episodes	19 (17)
3 or more episodes	6 (5)
Months duration (all episodes), median (IQR)	5 (3.75–7)
Bone marrow edema, *n* (%) of patients with MRI	94 of 103 (91)
Clinical fractures, *n* (%)	14 (13)^b^

^a^Not all publications provided precise reports on recurrent episodes, which introduces a minor risk of omissions in the data presented.

^b^All fractures were reported in perinatal women.

The ages in the aggregate‐level sample were somewhat higher, with the following means and SDs reported: 37 (NA) for *n* = 5, 49.14 (19.41) for *n* = 14, and 49.5 (16.7) for *n* = 65 (Note: The subpopulation used in this review consisted of 57/65). There were ≈ 58% women in the aggregate‐level sample, and none of them were perinatal.

A total of 13 patients, all perinatal women, were reported with fractures: 11 in the proximal femur, one in the sacrum, and one with thoraco‐lumbar compression fractures.

### 4.1. Individual Level BMD Data

In 87 patients, including 30 perinatal women, individual‐level DXA results were reported using at least one standardized BMD score. The scores included *Z*‐scores (*n* = 29), Z‐PEMs (*n* = 5), *T*‐scores (*n* = 52), and T‐PEMs (*n* = 1). A standardized hip score was available for 57 patients, a spine score for 67, and both for 39 patients. Hip results are presented in Table 3, prioritized in the following order: *Z*‐scores, *Z*‐PEMs, T‐scores, and T‐PEMs. Spine results are presented similarly in Table 4. Sixteen patients had standardized scores from both symptomatic and asymptomatic hips, while another 19 patients had raw DXA score results from both sides.

**TABLE 3:** DXA hip BMD (individual‐level data, *n* = 59).

BMD scores	Males (*n* = 27)		Nonperinatal women (*n* = 4)		Perinatal women (*n* = 28)	
	Symptomatic	Asymptomatic	Symptomatic	Asymptomatic	Symptomatic	Asymptomatic
*Z-scores (n = 27)*						
Hip						
Femoral neck	−1.9 (1.0) [7]	−0.2 (0.8) [7]	−1.3 (1.1) [2]	−2.8 (NA) [1]	−2.1 (0.4) [4]	
Total hip	−1.4 (1.2) [5]	−0.8 (0.6) [4]			−1.8 (0.8) [2]	
Unspecified	−2.6 (NA) [1]				−1.9 (1.1) [7]	−2.3 (1.5) [4]

Percent of expected mean for age and sex (Z‐PEMs) (n = 5)*^a^*						
Hip						
Femoral neck	87% (11) [3]	106% (12) [3]				
Total hip	63% (NA) [1]					
Unspecified	57% (NA) [1]					

T‐scores (n = 26)*^b^*						
Hip						
Femoral neck	−2.0 (0.7) [6]	−1.2 (NA) [1]	−1.4 (NA) [1]	−0.4 (NA) [1]	−2.7 (0.7) [7]	−2.6 (0.1) [2]
Total hip					−2.7 (0.9) [3]	−3.3 (NA) [1]
Unspecified	−2.3 (0.5) [5]	−1.8 (0.5) [5]	−2.4 (NA) [1]		−2.9 (1.4) [4]	−1.8 (NA) [1]

Percent of expected mean for young adults (T‐PEMs) (n = 1)*^c^*						
Hip						
Femoral neck						82% [1]

*Note:* Data are shown in prioritized order as mean (SD) (*n*) if not otherwise stated.

^a^In patients where *Z*‐scores were not reported.

^b^In patients where neither *Z*‐scores nor *Z*‐PEMs were reported. Only *T*‐scores for patients aged < 50 years were included.

^c^In a patient with no other standardized BMD scores.

**TABLE 4:** DXA spine BMD (individual‐level data, *n* = 67).

BMD scores	Males (*n* = 28)	Nonperinatal women (*n* = 16)	Perinatal women (*n* = 23)
Z‐scores (*n* = 19)	−0.9 (1.1) [7]	0.3 (NA) [1]	−2.3 (1.6) [11]
Percent of expected mean for age and sex (Z‐PEMs) (*n* = 1)^a^	81% (NA) [1]		
T‐scores (*n* = 46)^b^	−2.0 (0.8) [20]	−1.7 (0.8) [15]	−2.1 (0.7) [11]
Percent of expected mean for young adults (T‐PEMs) (*n* = 1)^c^			75% [1]

*Note:* Data are shown in prioritized order as mean (SD) (*n*) if not otherwise stated.

^a^In patients where *Z*‐scores were not reported.

^b^In patients where neither *Z*‐scores nor percentages of expected mean for age and sex were reported. Only *T*‐scores for patients aged < 50 years were included.

^c^In a patient with no other standardized BMD scores.

**FIGURE 2:**
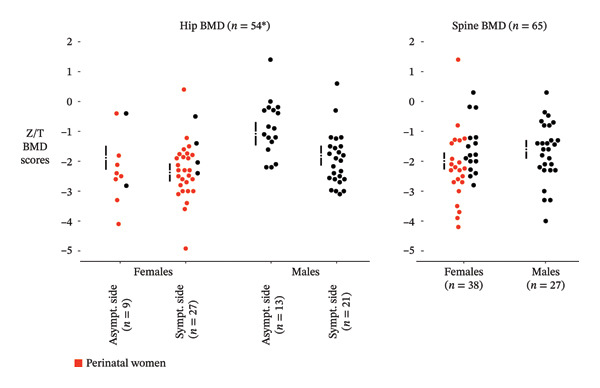
BMD *Z*‐ or *T*‐scores in asymptomatic and symptomatic hips and the lumbar spine. *Z*‐scores were preferred to *T*‐scores. Only *T*‐scores for patients aged < 50 years were included. The dots show the observed values from BMD measurements. The corresponding error‐bar to their left reflects the adjusted marginal means with 95% confidence intervals. As some patients had multiple measures, the hip marginal means were estimated post hoc from mixed‐effects model regression to account for the correlation, with all within‐patient measures taken concurrently. Red dots represent measurements in perinatal women. ^∗^54 patients with 83 measures.

The overall estimated marginal mean *Z*/*T*‐score was −2.12 (95% CI −2.4 to −1.88, *n* = 48) in symptomatic hips compared to −1.4 (95% CI −1.7 to −1.0, *n* = 22) in asymptomatic hips. Among females, the marginal mean *Z/T*‐score was −2.4 (95% CI −2.7, −2.1, *n* = 27) in symptomatic hips and −1.9 (95% CI −2.3 to −1.5, *n* = 9) in asymptomatic hips. Perinatal women accounted for 87% (27/31) of the females with hip *T/Z*‐scores. In males, the corresponding values were −1.8 (95% CI −2.1 to −1.5, *n* = 21) and −1.1 (95% CI −1.5, −0.7, *n* = 13) for symptomatic and asymptomatic hips, respectively (Figure 2).

**FIGURE 3:**
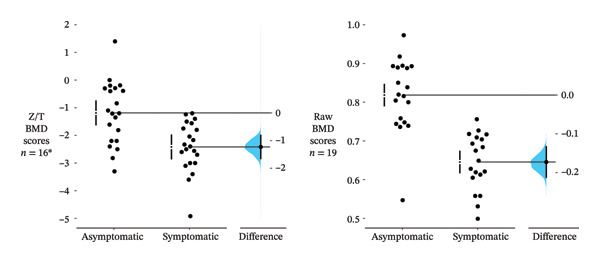
Gardner–Altman plots of differences between BMD scores in symptomatic and asymptomatic hips. The left panel shows results from *Z*‐ or *T*‐score data (*n* = 16). *Z*‐scores were preferred to *T*‐scores. Only *T*‐scores for patients aged < 50 years were included. The right panel shows results from raw BMD score data (*n* = 19). The dots show the observed values. The vertical error‐bars reflect the adjusted marginal means and 95% confidence intervals. The *Z*/*T* scores were estimated post hoc from mixed‐effects model regression to account for the correlation between multiple hip measurement sites, with all within‐patient measures taken concurrently. The separate scales to the right, horizontally aligned with the mean of the asymptomatic group, show the modelled mean difference Δ (asymptomatic − symptomatic) given the observed data with 95% confidence intervals. The blue curves show the resampled distribution of Δ, given the observed values. ^∗^21 measures for 16 patients.

Among patients with measurements from both symptomatic and asymptomatic hips, the estimated marginal mean *Z*/*T*‐score was 1.2 SDs lower in the symptomatic hips (95% CI −1.7 to −0.8, *n* = 16, Figure 3). The marginal mean BMD raw score was 0.173 g/cm^2^ lower in the symptomatic hips (95% CI −0.214 to −0.132, *n* = 19), corresponding to a 21% difference.

In the spine, the overall marginal mean *Z*/*T*‐scores was −1.83 (95% CI −2.1 to −1.6, *n* = 65). For females, the mean was −2.0 (95% CI −2.3 to −1.7, *n* = 38), and for males −1.6 (95% CI: −1.9 to −1.3, *n* = 27). Among perinatal women specifically, the estimated marginal mean *Z*/*T*‐score was −2.2 (95% CI −2.6 to −1.8, *n* = 22).

*Z*‐PEMs were reported only for male patients, with results consistent with the estimated marginal mean *Z*‐and *T*‐scores. One perinatal woman had *T*‐PEM values of 82% in the asymptomatic hip and 75% in the spine.

Among patients categorized under osteoporosis‐related terminology (*n* = 46), the marginal mean BMD in the symptomatic side was −2.1 (95% CI −2.4 to −1.9), compared to −1.8 (95% CI −2.2 to −1.5) in patients categorized under bone marrow edema‐related terminology (*n* = 7). In the asymptomatic side, the corresponding values were −1.4 (95% CI −1.7 to −1.0) and −1.5 (95% CI −1.8 to −1.1), respectively. One patient was categorised under “other” terminology.

Three patients, all postpartum females, were reported with standardized BMD scores obtained using modalities other than DXA: a lumbar QCT *Z*‐score of −1.8, a calcaneal QUS *Z*‐score of −3.4 and an SPA distal radius *Z*‐score of −3.4.

Statistical and visual assessments indicated a normal distribution of individual‐level BMD *Z*‐and *T*‐scores, supporting the representativeness of the data (Supporting 2.0).

### 4.2. Aggregate Level BMD Data

A total of 76 patients had aggregate‐level BMD data. No aggregate‐level *Z*‐scores were reported for symptomatic hips. For asymptomatic hips, the mean total hip *Z*‐score was −0.5 (SD 1.4, *n* = 14), and the mean adjusted spine *Z*‐score was −0.9 (SD 1.5, *n* = 71). *T*‐scores were reported for five patients, with mean values of −2.2 for the symptomatic total hip, −0.8 for the asymptomatic total hip, and −1.6 for the spine. Measures of variability for these *T*‐scores were not provided. Details regarding the location, recurrence of episodes, and duration in the aggregate‐level data were not sufficiently specific to allow for comprehensive analysis.

### 4.3. Change in BMD Over Time

**FIGURE 4:**
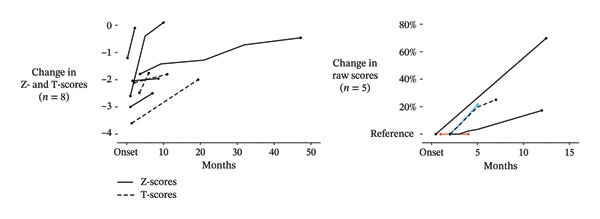
Change in bone density over time. Left panel: Changes in *Z*‐scores (solid lines) and *T*‐scores (dashed lines) (*n* = 8) from the time of symptom onset. Right panel: Percentage changes in raw scores (*n* = 5), with the reference set at onset. Vertical change indicates percentage improvement. Colored and dashed lines are used to differentiate individual trajectories.

Follow‐up BMD data for symptomatic hips were reported for 13 patients: 8 with *Z*‐ or *T*‐scores and 5 with raw scores. Except for two patients with short follow‐up periods, BMD generally increased over time (Figure 4). The increase in BMD coincided with clinical improvement in all but one patient. However, the small sample size, substantial interindividual variations in BMD changes, and inconsistencies in the reporting of clinical outcomes precluded analysis of the association between BMD changes and clinical improvement.

## 5. Discussion

This review is the first to compile the available data on BMD in patients with TOH, RMO, BMES, and similar designations. The findings indicate significantly reduced BMD in symptomatic hips relative to what would be expected for age and sex, with the reduction most pronounced in perinatal women but also notable in males. The number of nonperinatal women was too small for meaningful analysis. While BMD in asymptomatic hips was also reduced, the decrease was less pronounced than in symptomatic hips. Although follow‐up data were limited, they indicated an increase in BMD over time, coinciding with clinical improvement in most patients.

The most surprising finding was that bone density in the lumbar spine was reduced to a degree comparable to that of symptomatic hips. Almost none of the included patients reported lower back pain, indicating that the spine measurements were taken from asymptomatic bones. This reduction was consistently observed across males, perinatal women, and nonperinatal women. Our findings suggest a more widespread effect on bone density than previously recognized. While a few authors have noted reduced spine BMD [25–27], the focus has predominantly been on symptomatic bone until now. The clinical implications remain uncertain. Except for one perinatal woman with thoracolumbar compression fractures, spinal fractures were not reported. Long‐term studies are needed to clarify any potential need for antiosteoporotic prophylaxis or treatment.

Additionally, this study demonstrates that BMD results were broadly similar in patients classified using either osteoporosis‐related or bone marrow edema‐related terminology, further supporting the notion of a common underlying pathology.

### 5.1. Strengths and Limitations

The main limitation of this review is the observational and retrospective nature of the included studies, as well as the limited number of studies that reported quantifiable BMD data. Only 4% (112/2924) of the total patients identified in the original search had quantifiable BMD data and were eligible for inclusion. Furthermore, no diagnostic or classification criteria exist for TOH, RMO, BMES, or their various derivatives, making it difficult to ensure consistency across studies. As a result, the generalizability of our findings to the broader patient population remains uncertain. However, statistical and visual evaluation indicates a normal distribution of the individual‐level BMD *Z*‐ and *T*‐scores included in our analyses, suggesting reasonable representativeness of the broader population. We previously discussed the strengths and limitations of the original search and extraction process [5, 6].

Practically all evaluated measurements were performed using DXA, which is routinely applied to the hip and lumbar spine. Consequently, BMD data from patients whose primary symptoms were in the knees, ankles, or feet are generally lacking. Approximately two‐thirds of the patients identified in the original search had hip involvement, leaving BMD data for affected knees, ankles, or feet largely unknown. Additionally, the reporting among various authors was inconsistent and often incomplete. Some studies provided data for both hips and the spine, while others reported only on symptomatic or asymptomatic hips or solely on the spine. The specific measurement site was frequently unclear—some reported results for the total hip and/or femoral neck, while others simply stated “the hip” without further specification. In our analyses, we used all available data and accounted for the correlation between measurements from multiple sites using mixed‐effects regression analysis.

Another challenge was the inconsistent format in which BMD was reported. Some studies used *Z*‐scores, others used *T*‐scores, and some reported percentages of expected means, such as *Z*‐PEMs or *T*‐PEMs, or raw scores in g/cm^2^, complicating a unified analysis. To partially address this issue, we supplemented the analyses of *Z*‐score data with *T*‐score data from patients under 50 years of age. In young adults, *T*‐scores and *Z*‐scores are virtually identical because they are derived from the same reference population [28]. By age 50, T‐scores are considered about 0.5 SD lower than *Z*‐scores [14]. The majority of T‐score data came from the spine, meaning our estimates of spinal BMD may be slightly lower than if *Z*‐scores had been available for all patients. Another limitation is that the DXA measurements were performed over a span of more than 30 years, during which the definitions of *Z*‐ and *T*‐scores, their calculations across different technologies, and reference populations have varied [28–30]. For instance, *Z*‐scores are typically adjusted for age but may also be adjusted for gender, ethnicity, and weight [31]. This temporal and technological heterogeneity may have influenced observed BMD patterns; therefore, we caution against overinterpreting quantitative comparisons spanning 3 decades.

The relationship between perinatal BMES/TOH and pregnancy and lactation‐associated osteoporosis (PLO) remains unclear. It has been suggested that PLO results from altered calciotropic hormone levels or systemic bone resorption during pregnancy, whereas BMES/TOH is considered a focal disorder [32]. The findings of this study do not support this view. Spine and hip BMD in perinatal women are comparable to those reported in PLO [33, 34]. Note that this review did not include patients classified as PLO. None of the fracture patients included in this review had undergone densitometry of the hips that eventually fractured, making it impossible to determine if their BMD differed from hips that did not fracture.

### 5.2. Further Research

As documented here, the existing data on BMD in this patient group is sparse, inconsistent, and incomplete; hence, more research is strongly needed. To facilitate comparison of results across studies, we recommend that in future studies all data provided by DXA machines be reported, including information about the manufacturer’s reference norms used to compute standardized scores. The data should be accompanied by validated clinical outcome measures and standardized baseline data [35]. Long‐term follow‐up is especially desirable to determine whether and how long it takes for bone mineral content to return to normal and whether the condition increases the risk of fractures. Such knowledge will be important for treatment decisions.

## 6. Conclusion

This comprehensive literature review found evidence of substantially reduced BMD in symptomatic hips, asymptomatic hips, and the asymptomatic lumbar spine in patients with TOH, RMO, BMES, and similar conditions. However, the existing data are sparse and incomplete, highlighting the need for further research. Our findings support the notion that these terms reflect a common underlying pathology.

## Author Contributions

All authors conceptualized and designed the study, collected the data, and drafted the manuscript.

## Funding

The authors received no financial or material support for the research, authorship, and/or publication of this article.

## Disclosure

All authors have approved the manuscript after revising it critically for important intellectual content and gave final approval of the version to be published. Part of this work was submitted as an abstract and published in the proceedings of the Østfold Hospital Trust Research Seminar (*Forskningsseminar 2025*), Litteraturhuset Fredrikstad, June 5, 2025.

## Ethics Statement

This review did not require ethical approval as it involved the synthesis of publicly available literature without direct interaction with human participants.

## Conflicts of Interest

The authors declare no conflicts of interest.

## Supporting Information

This supporting material provides additional information and data related to the study presented in the main manuscript. It includes:

1. Patient counts broken down by the specific terminology used in the publications included in this scoping review
2. The statistical and visual checks used for the normality evaluation of BMD *Z*‐ and *T*‐scores.
3. A complete list of all 71 publications included in the review, categorized by those providing individual‐level and aggregate‐level data.
4. The completed Preferred Reporting Items for Systematic Reviews and Meta‐Analyses Extension for Scoping Reviews (PRISMA‐ScR) Checklist.

This Supporting Information is essential for providing the full context and rigorous methodological documentation of the review, offering further evidence for the data synthesis and supporting the conclusions of the main manuscript.

## Supporting information


**Supporting Information** Additional supporting information can be found online in the Supporting Information section.

## Data Availability

The data that support the findings of this study are available from the corresponding author upon reasonable request.
