# Supplementary material for: BMD in Transient Osteoporosis and Bone Marrow Edema Syndrome: A Scoping Review
Source: J Osteoporos. 2026 Apr 2;2026:9976282. doi: 10.1155/joos/9976282 (PMC13045316; doi:10.1155/joos/9976282)
Supplement: Supplementary file 1 — Supporting Information Additional supporting information can be found online in the Supporting Information section. [file JOOS-2026-9976282-s001.pdf]

Supplementary material

Bone mineral density in patients with transient osteoporosis of the hip, regional migratory osteoporosis, bone marrow edema syndrome, and related terms: A scoping review

Table of contents

1.0 Patient counts by terminology 2

2.0 Normality evaluation of BMD Z- and T-scores 3

3.0 Sources used in the review 4

3.1 Visualization of publication count sorted by year 4

3.2 List of publications (n = 68) with individual-level data (sorted by year) 4

3.3 List of publications with aggregate-level data (n = 3) 8

4.0 PRISMA-ScR Checklist 9

1.0 Patient counts by terminology

Counts shown by the total number of patients and for data collected at an individual-level and at an aggregate-level

Terminology*	Total	Individual level	Aggregate level
**Osteoporosis related terms**	**108**	**103**	**5**
Transient osteoporosis of the hip	75	75	-
Pregnancy related transient osteoporosis	15	15	-
Regional migratory osteoporosis	4	4	-
Other osteoporosis-related terms	14	9	5
**Bone marrow edema-related terms**	**80**	**9**	**71**
Bone marrow edema syndrome	75	4	71
Other bone marrow edema-related terms	3	3	-
Miscellaneous terms	2	2	-

* See the following publications for background regarding the terminologies:

Grøvle et al. The terminologies of transient, migratory, or localized osteoporosis, and bone marrow edema syndrome: a scoping review. Osteoporos Int 2024;35:217–26.

Hasvik et al. Clinical characteristics of patients with bone marrow edema syndrome, transient osteoporosis or migratory osteoporosis: a scoping review. Bone 2025.

2.0 Normality evaluation of BMD Z- and T-scores

Both the statistical normality tests and visual checks support approximate normality of the BMD Z- and T-scores reported in the studies population.

2.1 Normality tests of Z- and T-scores

Statistical test	Test statistic
Shapiro-Wilk normality test	$W = 0.986$, $p = 0.130$
Anderson-Darling normality test	$A = 0.743$, $p = 0.052$
D'Agostino skewness test	Skew = 0.315, $z = 1.601$, $p = 0.109$
Anscombe-Glynn kurtosis test	Kurt = 3.580, $z = 1.499$, $p = 0.134$

R, including the packages *nortest* 1.0–4 and *moments* 0.14.1 were used for analyses.

2.2 Visual checks of Z- and T-scores

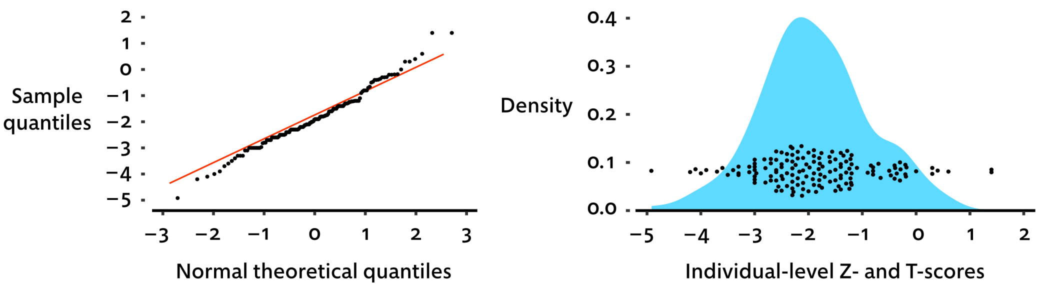

The left panel displays a Q-Q plot, comparing observed Z- and T-scores versus theoretical quantiles from the normal distribution, providing a visual assessment of normality. The red line indicates perfect normality.

The right panel presents a probability density plot, estimated with linear binning and the default oversmoothed bandwidth, using the *KernSmooth::bkde* function (v2.23-26). Individual patient scores are represented as dots, with jitter added on the y-axis to enhance separation. This visualization highlights the overall distribution of scores and reveals any potential gaps or skewness in the data.

Both figures include all Z- and T-score values for both hip and spine, from individual-level data, not including follow-up scores. It is important to note that this comprises multiple data points from some patients, which may influence the distribution.

R, including the packages *ggalt* 0.4.0, *ggbeeswarm* 0.7.2, *ggplot2* 3.5.1 was used for the plots.

3.0 Sources used in the review

3.1 Visualization of publication count sorted by year

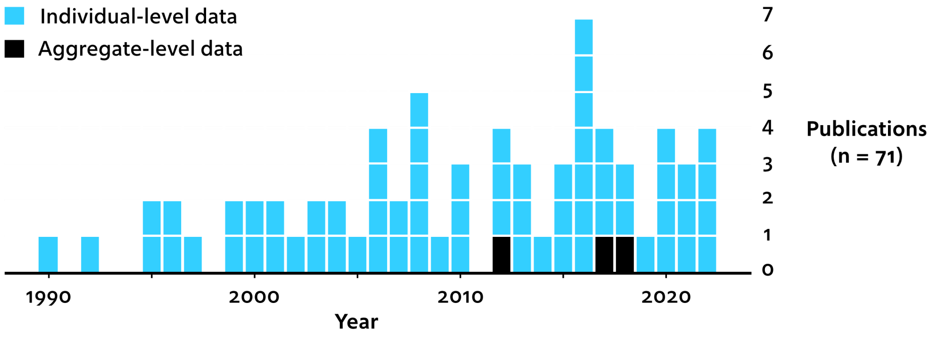

3.2 List of publications (n = 68) with individual-level data (sorted by year)

Banas, M. P.; Kaplan, F. S.; Fallon, M. D.; Haddad, J. G. (1990). Regional migratory osteoporosis: A case report and review of the literature. Clinical Orthopaedics and Related Research, Vol. 250, pp. 303-309 https://doi.org/http://dx.doi.org/10.1097/00003086-199001000-00040

Teruya, Tsutomu; Uesato, Tomomi; Mori, Satoshi; Nagayama, Moritaka; Ibaraki, Kunio; Nakasone, Satoshi (1992). Two Cases of Idiopathic Transient Osteoporosis of the Hip -Follow up Study with MRI. Orthopedics & Traumatology, Vol. 41, No. 2, pp. 627-631 https://doi.org/10.5035/nishiseisai.41.627

Carbone, Laura D; Palmieri, Genaro MA; Graves, Stanley C; Smull, Katherine (1995). Osteoporosis of pregnancy: long-term follow-up of patients and their offspring. Obstetrics & Gynecology, Vol. 86, No. 4, pp. 664-666

Funk, J. L.; Shoback, D. M.; Genant, H. K. (1995). Transient osteoporosis of the hip in pregnancy: Natural history of changes in bone mineral density. Clinical Endocrinology, Vol. 43(3), pp. 373-382 https://doi.org/http://dx.doi.org/10.1111/j.1365-2265.1995.tb02046.x

Varenna, M.; Sinigaglia, L.; Binelli, L.; Beltrametti, P.; Gallazzi, M. (1996). Transient osteoporosis of the hip: A densitometric study. Clinical Rheumatology, Vol. 15(2), pp. 169-173 https://doi.org/http://dx.doi.org/10.1007/BF02230335

Paran, D.; Segal, R.; Jukovsky, G.; Fishel, B.; Yaron, M. (1996). Transient regional osteoporosis with antiphospholipid antibodies: A report of two cases. Clinical and Experimental Rheumatology, Vol. 14(6), pp. 677-680

Stevens, R. J.; Hall, M. L.; Hughes, R. A. (1997). Imaging in transient regional osteoporosis [2]. British Journal of Rheumatology, Vol. 36(6), pp. 705-706

Maalouf, G.; Zein El Abidine, F.; Salem, S.; Bitar, F. (1999). Pregnancy associated osteoporosis: A case report and review of literature. [French]. Revue Medicale Libanaise, Vol. 11(1), pp. 20-23

Kato, Masaki; Iga, Kouzou; Kuwabara, Masaaki; Matsumoto, Kenji; Kawatani, Yosiyuki; Okumura, Hideo; Shibata, Taihoh (1999). Transient Osteoporosis of the Hip: A Report of Four Cases. The Journal of the Chugoku-Shikoku Orthopaedic Association, Vol. 11, No. 2, pp. 245-250 https://doi.org/10.11360/jcsoa1989.11.245

Sweeney, A. T.; Blake, M. A.; Holick, M. F. (2000). Transient osteoporosis of hip in pregnancy. Journal of Clinical Densitometry, Vol. 3(3), pp. 291-297 https://doi.org/http://dx.doi.org/10.1385/JCD:3:3:291

Lubbe, K.; Riedel, H. (2000). Transient regional osteoporosis during pregnancy. [German]. Geburtshilfe und Frauenheilkunde, Vol. 60(7), pp. 380-385 https://doi.org/http://dx.doi.org/10.1055/s-2000-7387

Cullu, E.; Savk, S. O.; Ozkan, I.; Alparslan, B. (2001). Transient osteoporosis of the hip joint; MRI, scintigraphy and bone mineral density assessment. [Turkish]. Artroplasti Artroskopik Cerrahi, Vol. 12(1), pp. 83-86

Bietenhard, K.; Altermatt, M.; Hohl-Korec, M.; Theiler, R. (2001). Transient osteoporosis during pregnancy. [German]. Praxis, Vol. 90(18), pp. 805-807

Varenna, M.; Zucchi, F.; Binelli, L.; Failoni, S.; Gallazzi, M.; Sinigaglia, L. (2002). Intravenous pamidronate in the treatment of transient osteoporosis of the hip. Bone, Vol. 31(1), pp. 96-101 https://doi.org/http://dx.doi.org/10.1016/S8756-3282%2802%2900812-8

Schapira, D.; Moscovici, Y. B.; Gutierrez, G.; Nahir, A. M. (2003). Severe transient osteoporosis of the hip during pregnancy. Successful treatment with intravenous biphosphonates. Clinical and Experimental Rheumatology, Vol. 21(1), pp. 107-110

Arayssi, T. K.; Tawbi, H. A.; Usta, I. M.; Hourani, M. H. (2003). Calcitonin in the treatment of transient osteoporosis of the hip. Seminars in Arthritis and Rheumatism, Vol. 32(6), pp. 388-397 https://doi.org/http://dx.doi.org/10.1053/sarh.2002.50014

Leistedt, S.; De Marneffe, P.; Burette, J. L.; Cornette, M. (2004). Image of the month: Bilateral spontaneous fracture of the femur neck with transient osteoporosis during pregnancy. [French]. Revue Medicale de Liege, Vol. 59(11), pp. 622-623

Guardiano, S. A.; Katz, J.; Schwartz, A. M.; Brindle, K.; Curiel, R. (2004). Fracture complicating the bone marrow edema syndrome. Journal of Clinical Rheumatology, Vol. 10(5), pp. 269-274 https://doi.org/http://dx.doi.org/10.1097/01.rhu.0000141509.18395.3c

La Montagna, G.; Malesci, D.; Tirri, R.; Valentini, G. (2005). Successful neridronate therapy in transient osteoporosis of the hip. Clinical Rheumatology, Vol. 24(1), pp. 67-69 https://doi.org/http://dx.doi.org/10.1007/s10067-004-0957-9

Şendur, Ö Faruk; Gürer, Gülcan; İyiyapici, Ayşe (2006). Geçici kalça osteoporozunun bir erkek olgu eşliğinde irdelenmesi. Osteoporoz Dünyasından, Vol. 12, No. 1, pp. 15-17

Niimi, R.; Sudo, A.; Hasegawa, M.; Fukuda, A.; Uchida, A. (2006). Changes in bone mineral density in transient osteoporosis of the hip. Journal of Bone and Joint Surgery - Series B, Vol. 88(11), pp. 1438-1440 https://doi.org/http://dx.doi.org/10.1302/0301-620X.88B11.18063

Kalliakmanis, A. G.; Pneumaticos, S.; Plessas, S.; Papachristou, G. (2006). Transient hip osteoporosis. Orthopedics, Vol. 29(3), pp. 263-264 https://doi.org/http://dx.doi.org/10.3928/01477447-20060301-15

Chowdhury, F. U.; Robinson, P.; Grainger, A. J.; Harris, N. (2006). Transient regional osteoporosis: A rare cause of foot and ankle pain. Foot and Ankle Surgery, Vol. 12(2), pp. 79-83 https://doi.org/http://dx.doi.org/10.1016/j.fas.2006.01.001

Bahadir, Cengiz; Gürel, Özbil Korkmaz; Ocak, Feride; Yiğit, Semra (2007). Kalçanın geçici osteoporozu: olgu sunumu. Osteoporoz Dünyasından, Vol. 13, No. 1, pp. 19-22

Steib-Furno, S.; Mathieu, L.; Pham, T.; Armingeat, T.; Porcu, G.; Gamerre, M.; Chagnaud, C.; Lafforgue, P. (2007). Pregnancy-related hip diseases: Incidence and diagnoses. Joint Bone Spine, Vol. 74(4), pp. 373-378 https://doi.org/http://dx.doi.org/10.1016/j.jbspin.2006.12.001

Ikemura, Satoshi; Yamamoto, Takuaki; Jingushi, Seiya; Nakashima, Yasuharu; Mawatari, Taro; Iwamoto, Yukihide (2008). Recurrent transient osteoporosis of the hip. European Journal of Radiology Extra, Vol. 66, No. 2, pp. e65-e69

Aynaci, Osman; Kerimoglu, Servet; Ozturk, Cagatay; Saracoglu, Metehan (2008). Bilateral non-traumatic acetabular and femoral neck fractures due to pregnancy-associated osteoporosis. Archives of orthopaedic and trauma surgery, Vol. 128, No. 3, pp. 313-316

Diwanji, S. R.; Cho, Y. J.; Xin, Z. F.; Yoon, T. R. (2008). Conservative treatment for transient osteoporosis of the hip in middle-aged women. Singapore Medical Journal, Vol. 49(1), pp. e17-e21

Bolland, M. J. (2008). Bilateral Transient Osteoporosis of the Hip in a Young Man. Journal of Clinical Densitometry, Vol. 11(2), pp. 339-341 https://doi.org/http://dx.doi.org/10.1016/j.jocd.2007.12.016

Karantanas, A. H.; Nikolakopoulos, I.; Korompilias, A. V.; Apostolaki, E.; Skoulikaris, N.; Eracleous, E. (2008). Regional migratory osteoporosis in the knee: MRI findings in 22 patients and review of the literature. European Journal of Radiology, Vol. 67(1), pp. 34-41 https://doi.org/http://dx.doi.org/10.1016/j.ejrad.2008.01.054

Pai, Wen-Chuan; Lin, Ching-Yang; Kao, Mu-Jung; Lin, Fong-Cheng (2009). Transient osteoporosis of the hip during pregnancy: A case report. Tw J Phys Med Rehabil, Vol. 37, No. 2, pp. 131-137

Guryel, Enis; Shaikh, Naveed; Clark, David W (2010). Displaced intracapsular fracture complicating transient osteopoenia of the hip in pregnancy: timing of surgery. Acta Orthopaedica Belgica, Vol. 76, No. 4, pp. 555

Kaya, E.; Ozyurek, S.; Zeki Kiralp, M. (2010). Transient hip osteoporosis. [Turkish]. Osteoporoz Dunyasindan, Vol. 16(1), pp. 29-30

Kalender, A. M.; Dogan, A.; Cakar, A.; Turkoz, T. (2010). Neglected bilateral femoral neck fracture associated with pregnancy and primary hyperparathyroidism. Acta Orthopaedica Belgica, Vol. 76(4), pp. 559-563

Truszczynska, A.; Walczak, P.; Rapala, K. (2012). Transient Peripartum Osteoporosis of the Femoral Head in First and Third Pregnancy. Journal of Clinical Densitometry, Vol. 15(4), pp. 467-471 https://doi.org/http://dx.doi.org/10.1016/j.jocd.2012.02.010

Pallavi, P.; Padma, S.; Vanitha, A. S. D. (2012). Transient osteoporosis of hip and lumbar spine in pregnancy. Journal of Obstetrics and Gynecology of India, Vol. 62(1), pp. S8-S9 https://doi.org/http://dx.doi.org/10.1007/s13224-013-0355-9

Fabbriciani, G.; Pirro, M.; Manfredelli, M. R.; Bianchi, M.; Sivolella, S.; Scarponi, A. M.; Mannarino, E. (2012). Transient osteoporosis of the hip: Successful treatment with teriparatide. Rheumatology International, Vol. 32(5), pp. 1367-1370 https://doi.org/http://dx.doi.org/10.1007/s00296-010-1404-7

Sarica, Mehmet Akif; Türkbeyler, İbrahim Halil; Babacan, Taner; Tosun, Hacı Bayram; Bulut, Taner (2013). Kalçanın çift taraflı geçici bölgesel osteoporozu: olgu sunumu. Gaziantep Medical Journal, Vol. 19, No. 3, pp. 207-209

Zofkova, I.; Hrbac, J.; Dostal, J.; Sprindrich, J. (2013). Regional migrating osteoporosis - A case report. [Czech]. Vnitrni Lekarstvi, Vol. 59(9), pp. 841-845

Anai, T.; Urata, K.; Mori, A.; Miyazaki, F.; Okamoto, S. (2013). Transient osteoporosis of the hip in pregnancy associated with generalized low bone mineral density - A case report. Gynecologic and Obstetric Investigation, Vol. 76(2), pp. 133-138 https://doi.org/http://dx.doi.org/10.1159/000351564

Siebachmeyer, M.; Fenton, P.; Rai, H. S.; Selzer, G. (2014). Bone marrow edema syndrome of the proximal part of the femur following gastric bypass surgery: A case report. JBJS Case Connector, Vol. 4(1) (no pagination) https://doi.org/http://dx.doi.org/10.2106/JBJS.CC.L.00302

Yi, S. R.; Lee, Y. H.; Kim, H. M. (2015). Bilateral Bone Marrow Edema Syndrome of the Femoral Head with a Unique Onset: A Case Report. Hip & Pelvis, Vol. 27, No. 4, pp. 273-7 https://doi.org/https://dx.doi.org/10.5371/hp.2015.27.4.273

Sas, S.; Kocak, F. A.; Kurt, E. E.; Erdem, H. R.; Tuncay, F. (2015). Rare cause of hip pain: Transient osteoporosis of the hip-Two case reports. [Turkish]. Turk Osteoporoz Dergisi, Vol. 21(3), pp. 141-144 https://doi.org/http://dx.doi.org/10.4274/tod.40412

Klontzas, M. E.; Vassalou, E. E.; Zibis, A. H.; Bintoudi, A. S.; Karantanas, A. H. (2015). MR imaging of transient osteoporosis of the hip: An update on 155 hip joints. European Journal of Radiology, Vol. 84(3), pp. 431-436 https://doi.org/http://dx.doi.org/10.1016/j.ejrad.2014.11.022

Teeuwen-Mutter, J; Aff, R; Beck, M (2016). Fortschreitende Schmerzsymptomatik der unteren Extremität mit Immobilität in der Schwangerschaft. Der Gynäkologe, Vol. 49, No. 5, pp. 381-385

Haugen, Anne Julsrud; Rashid, Haroon Ur; Hasvik, Eivind; Gleditsch, Jostein; Grøvle, Lars (2016). En mann i 40-årene med smerter i høyre lår og kne. Tidsskrift for Den norske legeforening

Delen, Veysel; Hız, Özcan; Alpaycı, Mahmut; Ediz, Levent (2016). Transient Osteoporosis of the Hip and Sacral Insufficiency Fracture in a Pregnant: A Case Report. Causapedia

Tasci Bozbas, G.; Gurer, G.; Sendur, O. F.; Alkan, A. G. (2016). Migratory transient osteoporosis: Atypical migration to the bilateral knee after childbirth. Turkiye Fiziksel Tip ve Rehabilitasyon Dergisi, Vol. 62(2), pp. 178-181 https://doi.org/http://dx.doi.org/10.5606/tftrd.2016.01709

Pavlov-Dolijanovic, S.; Vujasinovic Stupar, N.; Milenkovic, R.; Koletic, V. (2016). Transient osteoporosis of the hip in pregnancy or early avascular necrosis as observed by magnetic resonance imaging. Osteoporosis International, Vol. 1), pp. S457-S458 https://doi.org/http://dx.doi.org/10.1007/s00198-016-3530-x

Okada, Y.; Tsukada, S.; Saito, M.; Tasaki, A. (2016). Simultaneous Bilateral Transient Osteoporosis of the Hip without Pregnancy. Case Reports in Orthopedics, Vol. 2016, pp. 8491461 https://doi.org/https://dx.doi.org/10.1155/2016/8491461

Berman, N.; Brent, H.; Chang, G.; Honig, S. (2016). Transient osteoporosis: Not just the hip to worry about. Bone Reports, Vol. 5, pp. 308-311 https://doi.org/http://dx.doi.org/10.1016/j.bonr.2016.10.004

Santoso, A.; Ingale, P. S.; Park, K. S.; Yoon, T. R. (2017). Migratory bone marrow edema syndrome of the hips: A case report. Malaysian Orthopaedic Journal, Vol. 11(3), pp. 56-58 https://doi.org/http://dx.doi.org/10.5704/MOJ.1711.006

Kasahara, K.; Kita, N.; Kawasaki, T.; Morisaki, S.; Yomo, H.; Murakami, T. (2017). Bilateral femoral neck fractures resulting from pregnancy-associated osteoporosis showed bone marrow edema on magnetic resonance imaging. Journal of Obstetrics and Gynaecology Research, Vol. 43(6), pp. 1067-1070 https://doi.org/http://dx.doi.org/10.1111/jog.13313

Holub, A.; Fuertes, J. M. (2017). Transient osteoporosis of the hip during the pregnancy. the importance of screeing and the early diagnosis during the rutinary pregnancy check-up. Osteoporosis International, Vol. 28(Supplement 1), pp. S630-S631 https://doi.org/http://dx.doi.org/10.1007/s00198-017-3950-2

Aykurt Karlibel, I.; Kasapoglu Aksoy, M.; Altan, L. (2018). Familial transient osteoporosis of the hip? On account of three cases from the same family. Erciyes Medical Journal, Vol. 40(1), pp. 45-49 https://doi.org/http://dx.doi.org/10.5152/etd.2018.17093

Alsaed, O.; Hammoudeh, M. (2018). Recurrent Migratory Transient Bone Marrow Edema of the Knees Associated with Low Vitamin D and Systemic Low Bone Mineral Density: A Case Report and Literature Review. Case Reports in Rheumatology Print, Vol. 2018, pp. 7657982 https://doi.org/https://dx.doi.org/10.1155/2018/7657982

Carriles Rivero, I.; Auba Guedea, M.; Chacon Cruz, E.; Manzour Sifontes, N.; Galofre, J. C. (2019). Transient osteoporosis of the hip during pregnancy. Endocrinologia, Diabetes y Nutricion, Vol. 66(9), pp. 588-591 https://doi.org/http://dx.doi.org/10.1016/j.endinu.2019.03.016

Pimenta, T.; Parada, F.; Rocha, J. A. (2020). Bone Marrow Edema: A Case of Regional Migratory Osteoporosis. American journal of physical medicine & rehabilitation, Vol. 99(5), pp. e60-e63 https://doi.org/http://dx.doi.org/10.1097/PHM.0000000000001210

Paiva, F.; Simoes, N.; Pereira, D.; Borralho, N. (2020). Idiopathic Transient Osteoporosis during Pregnancy - Report of a Clinical Case. Journal of Orthopaedic Case Reports, Vol. 9, No. 6, pp. 54-57 https://doi.org/https://dx.doi.org/10.13107/jocr.2019.v09.i06.1586

Mori, K.; Kumar, S.; Yalamanchi, A.; Balachandran, K.; Asirwatham, A. R.; Mahadevan, S. (2020). Pregnancy associated Osteoporosis - Transient Osteoporosis of Hip. Journal of Bone and Mineral Research, Vol. 35(SUPPL 1), pp. 118 https://doi.org/https://dx.doi.org/10.1002/jbmr.4206

Ciftci, S.; Dogu, B.; Terlemez, R.; Yilmaz, F.; Kuran, B. (2020). Transient Osteoporosis of the Hip: A Case Report. Sisli Etfal Hastanesi Tp Bulteni, Vol. 54, No. 4, pp. 505-507 https://doi.org/https://dx.doi.org/10.14744/SEMB.2019.26879

Wakaki, Yu; Watanabe, Kaori; Ota, Kuniaki; Suzuki, Rika (2021). A Fracture of the Femoral Head during Pregnancy that caused by Transient Osteoporosis of the Hip（TOH）. Journal of Japan Society of

Perinatal and Neonatal Medicine, Vol. 57, No. 1, pp. 167-174 https://doi.org/10.34456/jjspnm.57.1_167

Altun, A.; Askin, A. (2021). A rare cause of hip pain: Transient osteoporosis of hip. Erciyes Medical Journal, Vol. 43(6), pp. 622-623 https://doi.org/https://dx.doi.org/10.14744/etd.2021.97105

Al-Dourobi, K.; Corbaz, J.; Bauer, S.; Leumessi, E. N. (2021). Lower lumbar back pain occurring with transient hip osteoporosis: Complication of prolonged suffering and neck of femur fracture in a 24-year-old pregnant patient. BMJ Case Reports, Vol. 14(1) (no pagination) https://doi.org/http://dx.doi.org/10.1136/bcr-2020-238477

Varming, Adam; Nymark, Tine; Pedersen, Lasse; Viberg, Bjarke (2022). Hip fracture in a pregnant woman. Ugeskrift for Laeger, Vol. 184, No. 43, pp. V05220334-V05220334

Siva, S.; Liao, Q.; Abbas, A.; Periasamy, K. (2022). Bilateral atraumatic femoral neck fractures resulting from transient osteoporosis of the hip. BMJ Case Reports, Vol. 15, No. 10, pp. e247967 https://doi.org/https://dx.doi.org/10.1136/bcr-2021-247967

Varenna, M.; Crotti, C.; Bonati, M. T.; Zucchi, F.; Gallazzi, M.; Caporali, R. (2022). A novel mutation in collagen gene COL1A2 associated with transient regional osteoporosis. Osteoporosis International, Vol. 33(1), pp. 299-303 https://doi.org/https://dx.doi.org/10.1007/s00198-021-06135-7

Bhakta, A. (2022). Transient osteoporosis in the third trimester of pregnancy: A case report. Case Reports in Women's Health, Vol. 34 (no pagination) https://doi.org/https://dx.doi.org/10.1016/j.crwh.2022.e00400

3.3 List of publications with aggregate-level data (n = 3)

Ringe, J. D.; Farahmand, P. (2012). Denosumab in the treatment of painful disabling transient regional osteoporosis (TRO) of the hip. Osteologie, Vol. 21(1), pp. A46

Rolvien, T.; Schmidt, T.; Butscheidt, S.; Amling, M.; Barvencik, F. (2017). Denosumab is effective in the treatment of bone marrow oedema syndrome. Injury, Vol. 48(4), pp. 874-879 https://doi.org/http://dx.doi.org/10.1016/j.injury.2017.02.020.*

* This publication was supplemented with data contained in the letter "Denosumab in bone marrow oedema syndrome. Response to Letter to the Editor of Injury" by Rolvien et al. Injury, Volume 48, Issue 10, 2368

Oehler, N.; Mussawy, H.; Schmidt, T.; Rolvien, T.; Barvencik, F. (2018). Identification of vitamin D and other bone metabolism parameters as risk factors for primary bone marrow oedema syndrome. BMC Musculoskeletal Disorders, Vol. 19(1) https://doi.org/http://dx.doi.org/10.1186/s12891-018-2379-x

4.0 PRISMA-ScR Checklist

Preferred Reporting Items for Systematic reviews and Meta-Analyses extension for Scoping Reviews (PRISMA-ScR) Checklist

SECTION	ITEM	PRISMA-ScR CHECKLIST ITEM	REPORTED ON PAGE #
**TITLE**			
Title	1	Identify the report as a scoping review.	1
**ABSTRACT**			
Structured summary	2	Provide a structured summary that includes (as applicable): background, objectives, eligibility criteria, sources of evidence, charting methods, results, and conclusions that relate to the review questions and objectives.	2
**INTRODUCTION**			
Rationale	3	Describe the rationale for the review in the context of what is already known. Explain why the review questions/objectives lend themselves to a scoping review approach.	3–4
Objectives	4	Provide an explicit statement of the questions and objectives being addressed with reference to their key elements (e.g., population or participants, concepts, and context) or other relevant key elements used to conceptualize the review questions and/or objectives.	4
**METHODS**			
Protocol and registration	5	Indicate whether a review protocol exists; state if and where it can be accessed (e.g., a Web address); and if available, provide registration information, including the registration number.	4
Eligibility criteria	6	Specify characteristics of the sources of evidence used as eligibility criteria (e.g., years considered, language, and publication status), and provide a rationale.	4
Information sources*	7	Describe all information sources in the search (e.g., databases with dates of coverage and contact with authors to identify additional sources), as well as the date the most recent search was executed.	4
Search	8	Present the full electronic search strategy for at least 1 database, including any limits used, such that it could be repeated.	4
Selection of sources of evidence†	9	State the process for selecting sources of evidence (i.e., screening and eligibility) included in the scoping review.	4
Data charting process‡	10	Describe the methods of charting data from the included sources of evidence (e.g., calibrated forms or forms that have been tested by the team before their use, and whether data charting was done independently or in duplicate) and any processes for obtaining and confirming data from investigators.	4–5
Data items	11	List and define all variables for which data were sought and any assumptions and simplifications made.	4–5
Critical appraisal of individual sources of evidence§	12	If done, provide a rationale for conducting a critical appraisal of included sources of evidence; describe the methods used and how this information was used in any data synthesis (if appropriate).	NA
Synthesis of results	13	Describe the methods of handling and summarizing the data that were charted.	5–6
**RESULTS**			

SECTION	ITEM	PRISMA-ScR CHECKLIST ITEM	REPORTED ON PAGE #
Selection of sources of evidence	14	Give numbers of sources of evidence screened, assessed for eligibility, and included in the review, with reasons for exclusions at each stage, ideally using a flow diagram.	6–7
Characteristics of sources of evidence	15	For each source of evidence, present characteristics for which data were charted and provide the citations.	6–8
Critical appraisal within sources of evidence	16	If done, present data on critical appraisal of included sources of evidence (see item 12).	NA
Results of individual sources of evidence	17	For each included source of evidence, present the relevant data that were charted that relate to the review questions and objectives.	6–8
Synthesis of results	18	Summarize and/or present the charting results as they relate to the review questions and objectives.	6–8
**DISCUSSION**			
Summary of evidence	19	Summarize the main results (including an overview of concepts, themes, and types of evidence available), link to the review questions and objectives, and consider the relevance to key groups.	9
Limitations	20	Discuss the limitations of the scoping review process.	9–10
Conclusions	21	Provide a general interpretation of the results with respect to the review questions and objectives, as well as potential implications and/or next steps.	10–11
**FUNDING**			
Funding	22	Describe sources of funding for the included sources of evidence, as well as sources of funding for the scoping review. Describe the role of the funders of the scoping review.	1

JBI = Joanna Briggs Institute; PRISMA-ScR = Preferred Reporting Items for Systematic reviews and Meta-Analyses extension for Scoping Reviews.

* Where *sources of evidence* (see second footnote) are compiled from, such as bibliographic databases, social media platforms, and Web sites.

† A more inclusive/heterogeneous term used to account for the different types of evidence or data sources (e.g., quantitative and/or qualitative research, expert opinion, and policy documents) that may be eligible in a scoping review as opposed to only studies. This is not to be confused with *information sources* (see first footnote).

‡ The frameworks by Arksey and O'Malley (6) and Levac and colleagues (7) and the JBI guidance (4, 5) refer to the process of data extraction in a scoping review as data charting.

§ The process of systematically examining research evidence to assess its validity, results, and relevance before using it to inform a decision. This term is used for items 12 and 19 instead of "risk of bias" (which is more applicable to systematic reviews of interventions) to include and acknowledge the various sources of evidence that may be used in a scoping review (e.g., quantitative and/or qualitative research, expert opinion, and policy document).

From: Tricco AC, Lillie E, Zarin W, O'Brien KK, Colquhoun H, Levac D, et al. PRISMA Extension for Scoping Reviews (PRISMAScR): Checklist and Explanation. Ann Intern Med. 2018;169:467–473. doi: 10.7326/M18-0850.
